# Metopic synostosis

**DOI:** 10.1007/s00381-012-1803-z

**Published:** 2012-08-08

**Authors:** Jacques van der Meulen

**Affiliations:** Dutch Craniofacial Unit, Department of Plastic, Reconstructive and Hand Surgery, Sophia Children’s Hospital, Erasmus Medical Center, Dr Molewaterplein 60, 3015GJ Rotterdam, The Netherlands

**Keywords:** Craniosynostosis, Trigonocephaly, Review, Treatment, Neurodevelopment, Follow-up

## Abstract

Premature closure of the metopic suture results in a growth restriction of the frontal bones, which leads to a skull malformation known as trigonocephaly. Over the course of recent decades, its incidence has been rising, currently making it the second most common type of craniosynostosis. Treatment consists of a cranioplasty, usually preformed before the age of 1 year. Metopic synostosis is linked with an increased level of neurodevelopmental delays. Theories on the etiology of these delays range from a reduced volume of the anterior cranial fossa to intrinsic malformations of the brain. This paper aims to provide an overview of this entity by giving an update on the epidemiology, etiology, evolution of treatment, follow-up, and neurodevelopment of metopic synostosis.

## Introduction

**Fig 1:**
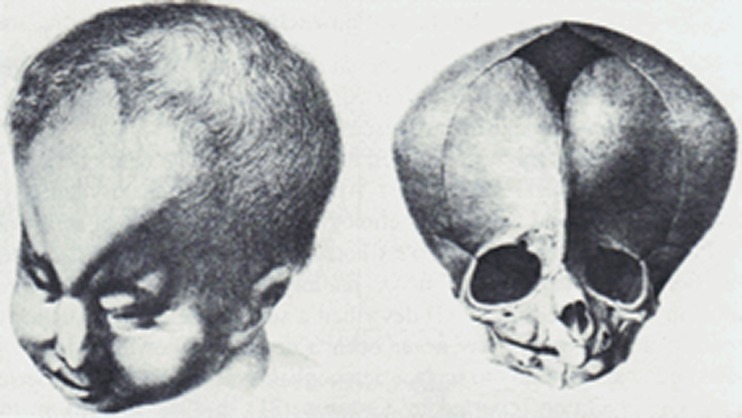
Metopic synostosis as described by Welcker in 1862

The term trigonocephaly is derived from the Greek words “*trigonon”*, which means triangle, and “*kephale*”, which means head. This type of craniosynostosis is thus characterised by a triangular, or wedge-shaped forehead, resulting from a premature fusion and subsequent ossification of the metopic suture (Greek “*metopon*” = forehead). The term trigonocephaly was first proposed by Welcker in 1862, who used it to describe a child presenting with a wedge-shaped skull combined with a cleft lip (Fig. 1) [105].

The metopic suture separates the two frontal bones at birth and is the first skull suture to close physiologically, starting as early as at 3 months and generally being completely fused at the age of 8 months [101, 104]. A premature fusion however, results not only in an obvious ridge over the midline of the forehead due to ossification of the suture, but also in a lateral growth restriction of the frontal bones. According to the theory of Virchow, this wedge shape is even further enhanced by the increased compensatory growth of the remaining skull sutures while the skull keeps expanding [100].

**Fig 2:**
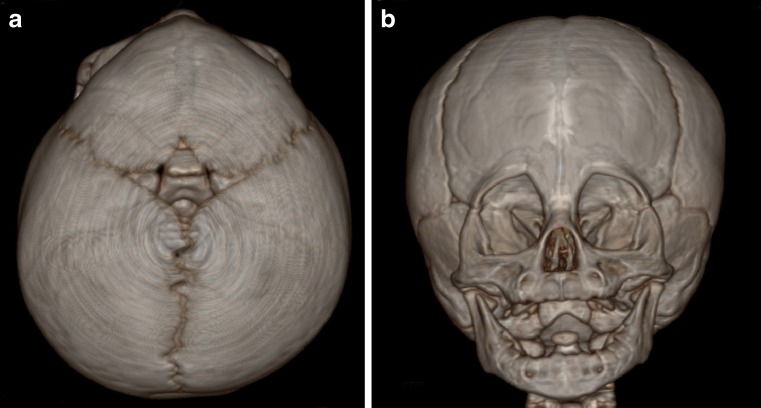
**a** CT scan of metopic synostosis (*top view*). **b** CT scan of metopic synostosis (*AP view*)

The end product is a skull with a triangular forehead, a bony midline ridge and a shortening of the anterior cranial fossa. (Fig. 2a). Often there is some degree of soft tissue excess along the same line. In 55 % of cases, the anterior fontanel is closed prematurely [18]. Deficient lateral orbital rims add to the supraorbital retrusion and the bitemporal indentations. In severe cases, the lateral canthal angles are elevated. At the level of the medial orbital walls, there is hypotelorism combined with ethmoidal hypoplasia. Epicanthal folds are often present. The orbits are teardrop shaped and angulated towards the midline of the forehead (Fig. 2b). Vertical growth restriction as expressed in reduced auricular head height is one of the most significant components of the midline growth anomalies. The cephalic index (maximal skull width/maximal skull length) remains within normal limits, even though there is bitemporal shortening and biparietal widening [6, 8, 15, 28, 30, 53, 57, 75, 81, 91, 109]. Since the growth restriction results in a reduced intracranial volume, surgery is indicated to restore the scull volume as well as its appearance.

## Clinical range of phenotype

The severity of metopic synostosis can vary considerably. The premordia of trigonocephaly can be seen in children with a metopic ridge due to an increased deposition of bone along the metopic suture. The etiology of this finding is unknown and usually there are no other clinical or radiological features. The supra-orbital retrusion, which is so typical in trigonocephaly, ranges from mild to severe and can be classified using the following methods:

## Frontal angle

**Fig 3:**
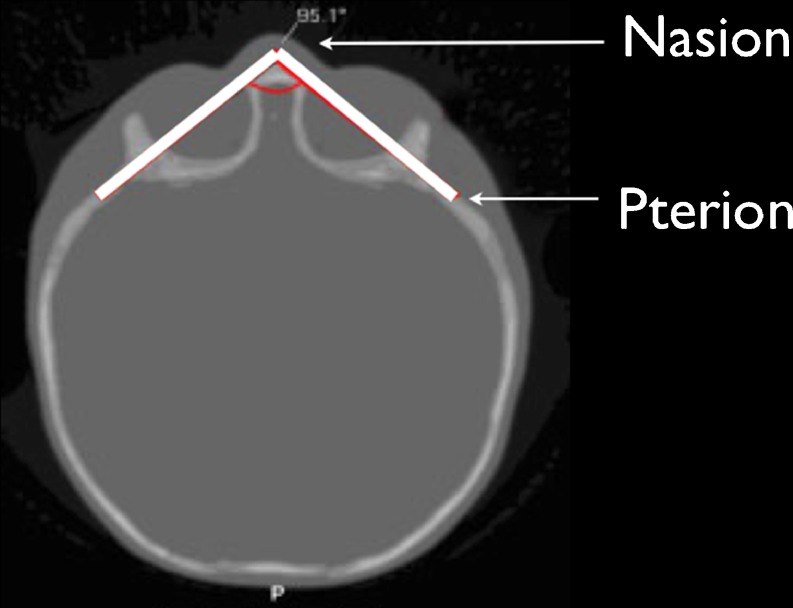
Frontal angle as described by Oi in 1986

The frontal angle is defined as the angle between the two lines drawn through Pterion (bilaterally) and Nasion, as described by Oi and Matsumoto in 1986 (Fig. 3). Measurements were done on axial CT slices. According to these calculations, a trigonocephaly was classified as being severe when presenting with an angle of less than 89°, moderate when between 90° and 95°, mild when between 96° and 103°, and normal when measuring 104° or more [72].

## Frontal stenosis

**Fig 4:**
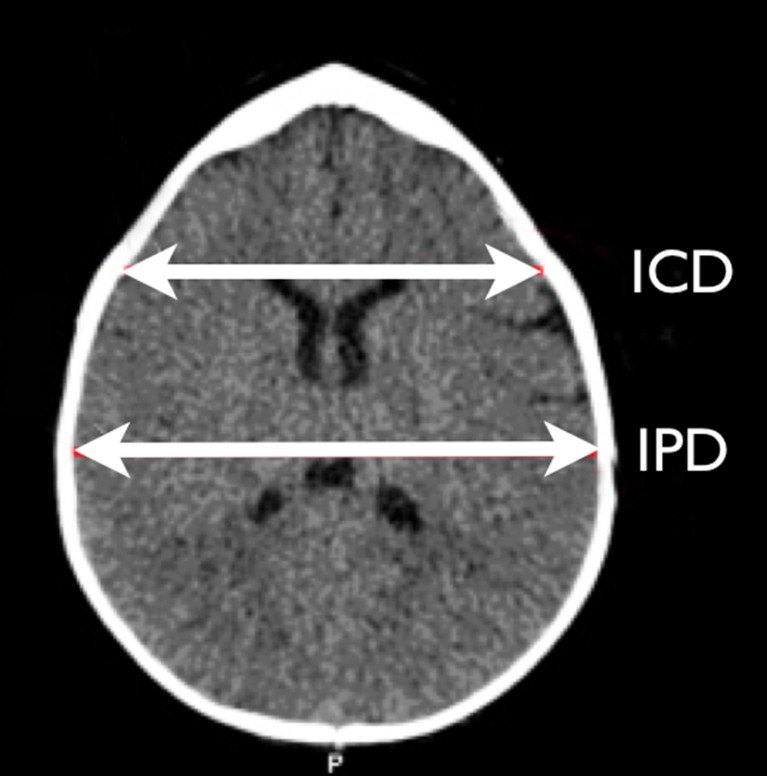
Frontal stenosis ratio as described by Bottero [13]

This is defined as the ratio of the interparietal distance to the intercoronal distance according to the method introduced by Posnick et al. in 1994 and further modified by Bottero et al. (Fig. 4) [13, 75]. Shimoji subsequently determined the IPD/ICD to be 1.21 in normal children [87]. Again, axial CT slices were used to perform the measurements.

Even though the majority of trigonocephaly cases appear to be confined to the metopic suture itself, syndromes occur in around 35 % of cases [57]. Syndromes associated with trigonocephaly are:

- Baller–Gerold [76, 93]
- Muenke [96]
- Saethre–Chotzen [20]
- Say–Mayer [83]
- Opitz C [36, 82]

## Epidemiology

The range of incidence of metopic synostosis has been reported to be rather wide, somewhere between 1:700 and 1:15,000 newborns [2, 57]. Traditionally, in series presenting an overview of more than 100 craniosynostotic cases, metopic synostosis used to account for 3 to 27 % of the total, making it the third most common single suture synostosis after sagittal and unicoronal synostosis [7, 28–35].

The incidence is on the rise though. A Pan-European study (seven units, 3,240 cases) published in 2008 revealed a significant increase of the absolute number as well as of the percentage of metopic synostosis over the period 1997 to 2006. The most remarkable increase occurred around 2000–2001, with the average of metopics being 20.1 % from 1997 to 2000 and 25.5 % from 2001 to 2005 [98]. Others have confirmed this observation, with Selber et al. reporting on a rise of metopic prevalence within their unit of 3.7 % in 1975 to 27.3 % in 2004 while Di Rocco et al. noted an increase of 420 % over 20 years [23, 24, 85]. These observations confirm that metopic synostosis is now the second most frequently seen type of craniosynostosis.

Taking advantage of the systematic national registration system in the Netherlands, we recently managed to provide a comprehensive overview of all craniofacial cases seen in this country over the last decade (927). This study put the incidence of metopic synostosis currently at 1:5,200 [56].

The male to female ratio is reported to be between 2:1 [25, 30, 33] and 6.5:1 [22], with Lajeunie et al. noting a ratio of 3.3:1 in the largest series to date (237 cases). They also found a positive family history in 10 out of the 179 families (5.6 %) and a 7.8 % frequency of twins. Fifty-three of their cases (22.4 %) were associated with other malformations (13 well-defined syndromic cases and 40 cases with one or more malformations but without a known syndrome) [57]. Shillito found associated abnormalities in 19 % of their 21 cases, with 9.5 % presenting with multiple abnormalities [86]. Boulet et al. reported that, in their study of 854 children, increased maternal age and a birth weight of less than 2,500 g was associated with a higher risk of metopic synostosis [14].

## Etiology

The etiology of metopic synostosis is largely unknown, but three theories predominantly arise:

1. Intrinsic bone malformation

The classical and most popular theory of premature suture fusion points towards osseous pathology early on in the pregnancy. This is believed to occur either by genetic [107, 108], metabolic [73], or pharmaceutical [57] means. In metopic synostosis especially, these different etiological factors are all represented. In one reported case, a fibroblast growth factor receptor 1 mutation was shown to be present in metopic synostosis [54]. Lajeunie et al. showed hereditary proof in 5.6 % of their cases [57], with others quoting the autosomal dominant penetration to be 2–5 % [31, 38]. Thyroid hormone replacement therapy in case of hypothyroidism has been shown to cause (metopic) craniosynostosis [48, 73, 77] as has been the case with the use of the anticonvulsant drug Valproate during pregnancy [9, 57]. The suggestion that folic acid is involved in the etiology of metopic synostosis is tempting but has yet to be proven [49, 85, 98].

2. Fetal head constrain

The second theory places the onset of the synostosis in the last phase of the pregnancy, when the head of the fetus can be constrained in the pelvic area. Graham and Smith described two cases of metopic synostosis believed to be the result of limited space for the fetal head (one was jammed in a bicornuate uturus, the other one between the legs of his two siblings) [34]. More recently this theory was supported by Smartt et al., proving the principle in a mouse model [90].

3. Intrinsic brain malformation

The third theory considers the brain to be the main reason behind the onset of craniosynostosis [68, 80]. The malformation of the frontal lobes would thus require only limited space in the anterior cranial vault, therefore providing a more restrained signal to the bone centres causing the suture to fuse prematurely. Findings of neurodevelopmental delays irrespective of corrective cranioplasty have further supported this theory [52].

A combination of the first and last theory could imply a genetic disorder, even though the usual candidates (FGFR1-3, TWIST, and EFNB1) have only occasionally been isolated in trigonocephaly [54, 96]. Metopic synostosis is however associated with several chromosomal disorders:

- 3q, 7p [44, 46]
- 9p22–24 [5, 46]
- 11q23 (Jacobsen syndrome) [11, 70]
- 22q11.2 [70]

There seems to be ample proof for all three theories to be able to safely conclude that the etiology of metopic synostosis is multifactorial.

## History of treatment

In 1921, the first report dealing with the surgical treatment of craniosynostosis appeared, when Mehner published his technique of removing the fused cranial suture [67]. This was to be the method of choice for years to come while the main problem appeared to be the prevention of early re-fusion of the suture [7, 86]. Matson subsequently published his technical notes on limited strip craniectomy in 6 cases of trigonocephaly in 1960, setting the standard for the next generation of (neuro)surgeons [64]. He commented that surgical correction for metopic synostosis was only of cosmetic value and only worth it if carried out in the first 4 months of life. Two years later, Anderson advocated doing a simple cranial vault procedure before the age of 3 months but only if the child was not retarded or suffering from other major anomalies like heart disorders [8]. In 1968, Shillito et al. reported on 519 cranioplasties preformed from January 1929 to December 1966 [86]. In the largest series to that date, they stimulated early operative treatment to “provide at minimal risk the best chances for the brain to expand the skull into its normal configuration”. This coincided with the publication of the pioneering work of Paul Tessier in 1967, making the surgical treatment of craniosynostosis and its sequelae more common practise [94].

## Recent evolutions of treatment

There has been one paper describing the natural history of trigonocephaly to be self-limiting, although nobody since has reported the same [26, 35, 109]. Treatment therefore is commonly accepted to be surgical. Due to claims of better intellectual outcome, the operative correction is generally performed before the age of one [6, 17, 22, 25, 61, 65, 79, 86, 106].

Simple suturectomy is nowadays considered to be insufficient to correct the complex three dimensional growth restrictions that result from metopic synostosis [6, 21, 30, 43, 60]. Hoffman and Mohr published a paper in 1976 on their technical notes regarding the correction of trigonocephaly, which involved the advancement of the lateral canthal segments of the supraorbital regions [43]. Marchac followed up in 1978 with his classic paper on correction of the forehead using the “floating forehead technique” combined with remodelling of the supra-orbital bandeau [60]. Several authors have since modified this technique [10, 17, 22, 25, 28, 30, 81, 84], some with emphasis on the prevention of postoperative temporal hollowing [1, 62, 63, 71, 74, 103]. Others have ventured into different directions in their quest to correct these deformities with minimal risk and maximal result. Distraction osteogenesis with conventional screws or with springs has been introduced and has been gaining wider acceptance over the last years, especially with regards to the correction of hypotelorism, even though there has been some debate whether this hypotelorism really needs to be corrected [29]. Some have noted the deformity to persist over the years [30, 75] while others have adjusted their operative techniques with success [37, 66, 84]. Nevertheless, the role of springs in moving the orbits apart has been explored with success [19, 58, 59]. The use of minimal invasive endoscopic surgery techniques is on the rise since the early 1990s but still controversial due to the technical limitations of those procedures (strip craniectomy only), although Hinojosa has recently attempted to address those limitations [12, 41, 42, 47, 69].

## Fronto-supra-orbital advancement and remodelling

### Author’s technique

Preoperatively all our patients are screened for papillary oedema. Standard radiographic workup consists of plain scull radiographs and a 3D CT scan (1 mm slices), which is used for confirmation of the diagnosis as well as evaluation of intracranial abnormalities. In 72 % or our cases intracranial abnormalities were found, the majority showing frontal hypoplasia and/or ventricular dilatations.

**Fig 5:**
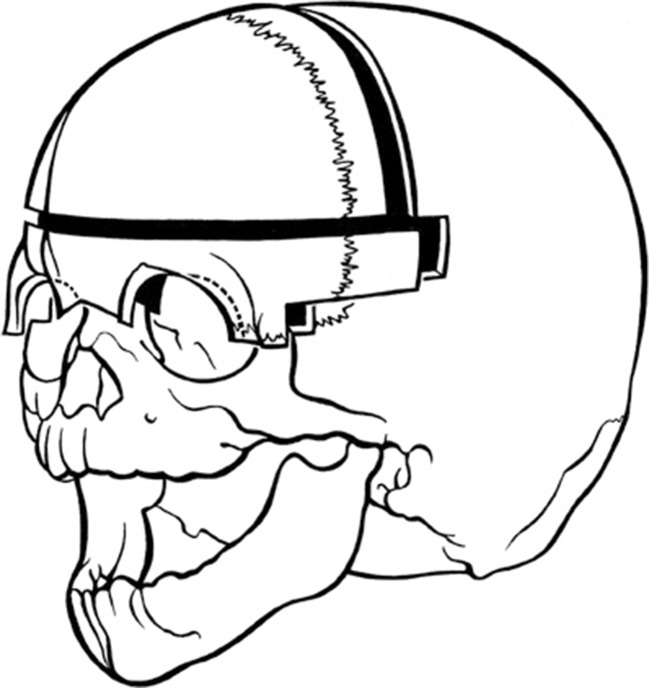
Fronto-supraorbital advancement (Author’s method)

The standard technique for the surgical correction of trigonocephaly performed at the National Craniofacial Center in the Sophia Children’s Hospital of the Erasmus Medical Center (Rotterdam, The Netherlands) is as follows:

After general anaesthesia, the patient is positioned in 20 degrees anti-Trendelenburg and a bicoronal, zigzag skin incision is used to provide access. The skin is mobilised together with the galea to 1–2 cm cranially of the supra-orbital rim. The periosteal layer is then mobilised separately. The superior half of the orbital content is loosened and the temporal muscles are freed from their cranial attachments. The frontal bone is removed in one piece, followed by the supra-orbital bar (Fig. 5). Meticulous haemostasis is achieved at this stage using bonewax.

**Fig 6:**
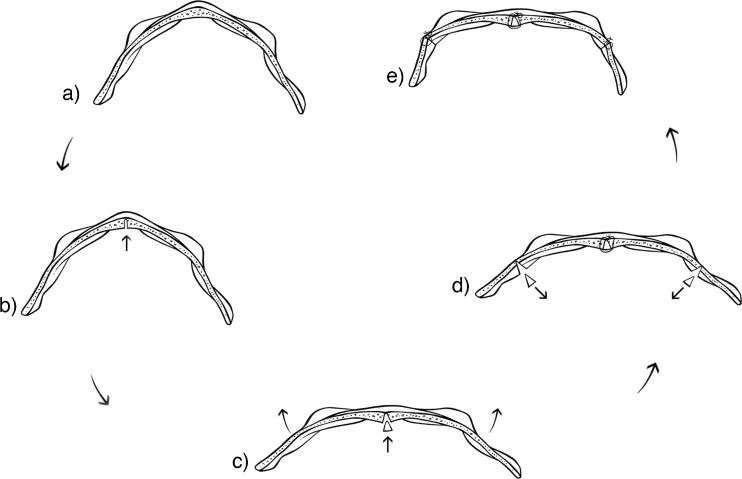
Remodellation of supraorbital bar (Author’s method)

The supra-orbital bar (Fig. 6a) is then addressed by an open wedge osteotomy, which is performed in the posterior midline. This facilitates bending the bar into a more horizontal position, therefore correcting the angle between the orbits (Fig. 6b). This movement increases the inter-orbital distance, thus eliminating the need for an interpositional bone graft. A unicortical posterior bone graft is subsequently used though to stabilise the midline open wedge osteotomy (Fig. 6c). A closed wedge osteotomy is performed lateral of the lateral orbital wall, by which an increase of the fronto-temporal angle is achieved (Fig. 6d, e). The temporal fragments of the bar are then moved forward in a “tongue-in-groove” fashion.

**Fig 7:**
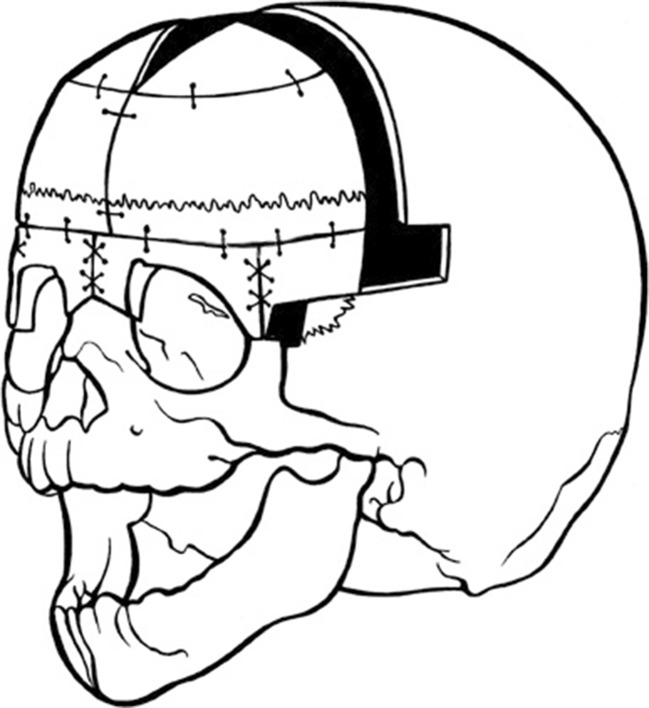
Fronto-supraorbital remodellation (Author’s method)

The frontal bone is cut in the midline and remodelled to fit to the new shape of the supra-orbital bar. This usually results in the two halves being switched and rotated 120°, so both coronal sutures end up parallel to the supra-orbital osteotomy line (Fig. 7). Absorbable sutures are used (2/0 and 3/0 Vicryl®, Polyglactine 910, Johnson & Johnson) to obtain fixation.

The fronto-supraorbital remodellation and advancement procedure thus manages to restore the volume of the anterior vault and corrects the morphological changes.

### Follow-up

Our patients are seen according to a follow-up protocol, commencing at 3 weeks after surgery for general wound inspection (see Table 1). Radiographs are taken at regular intervals, initially to evaluate postoperative re-ossification, but later on as part of the check for signs of raised intracranial pressure. Fundoscopy is considered to be a vital aspect of this screening, with a 100 % sensitivity for detecting raised intracranial pressure in children of 8 years and older [95].

**Table 1:** Follow-up schedule for metopic synostosis

Out patients evaluation	Scull circumference	Plain radiographs (AP and lateral)	Fundoscopy
Preoperatively	*X*	*X* ^a^	*X*
3 weeks postoperative	*X*	–	–
3 months postoperative	*X*	*X*	–^b^
At age 2 years	*X*	*X*	*X*
At age 4, 6, and 9 years	*X*	*X*	–^c^
At age 12 and 15 years	*X*	–	–^c^
At age 18 years	*X*	*X*	–^c^

^a^Preoperative screening includes a Tschebull radiograph and a 3D CT scan

^b^Only when preoperative fundoscopy showed papillary oedema

^c^Only when raised intra cranial pressure is suspected (inhibited cranial expansion, increased beaten copper pattern, changed behaviour, visual impairment, etc)

Patients are seen biannually from 2 till 6 and every 3 years from then onwards till the end of their growth process at the age of 18 years.

### Evaluation of aesthetic results

Anderson presented the results of 107 cases of metopic and coronal synostosis in 1981, advising “that craniofacial operations for synostosis should be as extensive as necessary” [6]. After that, Freide et al. were one of the first to attempt an aesthetic evaluation of their treatment for metopic synostosis [30]. Their retrospective review of 11 cases consisted of six operated and five non-operated children with metopic synostosis. Advancement and straightening of supraorbital bone contour was performed in all six cases. Three to four years after surgery, the osteotomy lines where hardly found on palpation except temporally where the tongue in groove advancement sometimes yielded slight bone irregularity. They concluded that, since minor characteristics were still present after such a long time, a modification seemed appropriate to enhance restitution of forehead width and morphology of the temporal regions. Cohen et al. noted none or minor irregularities in 9 of their 17 cases in which photographic analysis was done. Their reoperation rate was 18 % [16]. Posnick et al. investigated structural improvements of the periorbital region following corrective surgery using CT data in ten patients, concluding that “anterior cranial vault and lateral orbital wall positions were corrected successfully and remained in good position despite subsequent growth. The orbital hypotelorism, although improved, remained undercorrected” [75]. Havlik et al. adjusted their technique based on these same issues of correction of hypotelorism and prevention of temporal hollowing in ten cases with severe trigonocephaly, using a midline interposition bonegraft and temporal extension graft to reduce these problems [37]. They later on follow-up on this and reviewed their 68 metopic synostosis patients, concluding that preoperative frontal irregularities and reduced preoperative intercanthal distance predisposed to inferior aesthetic outcome while interpositional bonegrafting reduced the postoperative rate of temporal hollowing [37, 84]. In 2002, Hinojosa commented on their series of 28 cases, grading as high as 85 % good to excellent cosmetic results with an average follow-up of a little over 2 years (27 months) [40]. Aryan et al. noticed a recurrence of the midline ridge in 3 out of their 39 cases, requiring a reoperation in two [10]. Hilling et al. remarked that results were persistently good over the years if the operation managed to achieve good reposition of the forehead in the first place [39]. Greenberg et al. recently found a 15 % reoperation rate in their 50 cases, again mainly for correction of temporal hollowing [35].

An extensive radiological analysis of the largest series to date (92 cases, all operated according to the technique described above) revealed a tendency of auto-correction of the hypotelorism as a result of an increased postoperative interorbital growth rate. Temporal hollowing seemed to be the most commonly seen postoperative abnormality, which coincided with a notably reduced postoperative growth rate of the bony temporal region [97]. A subsequent study confirmed that reduced bone growth (and not soft tissue factors) was the major contributor to this temporal hollowing [99].

### Neuropsychological development

Of all the single suture synostoses, children with metopic synostosis have shown to be linked with the highest percentage of neurodevelopmental problems. Shillito et al., in their 1968 review of 519 cases, noted that “mental retardation was twice as high (4.8 %) compared with children with sagittal or coronal synostosis” [86]. Anderson in 1981 reported on a retardation rate of 17.9 % in their population of trigonocephalies [6]. Different authors have since described neurodevelopmental delays, ranging from 15 to as high as 61 % [10, 17, 72, 89]. Many of these problems do not become apparent until the children reach a school going age, where they are positioned into more intellectually demanding surroundings combined with higher expectancies of social interaction [50].

Elevated intra cranial pressure (ICP) has been linked to a reduction of IQ [45, 87, 88]. Levels of 8 to 20 % of elevated ICP in single-suture synostosis have been reported [27, 32, 78, 92]. Shillito et al. noted an increased ICP in 19 % of their 21 metopic cases, 18 of which were operated on. In their series this percentage was second only to the percentage in cases of multiple suture synostoses (41 %). They did not however directly measure the pressure: separation of uninvolved sutures on X-ray, the presence of a beaten copper pattern or papillary edema, and marked irritability (only if it disappeared after surgery) were considered to be signs of elevated ICP [86].

Although some authors have claimed to see no developmental effect whatsoever [30, 64], IQ inhibitions were reported by several units [72, 79], while others noticed the effects to largely be at the level of neurodevelopmental disorders [13, 16, 50–52, 55, 89, 102]. Boterro et al. for instance tested 76 children with metopic synostosis and showed developmental delay in 32 % of operated children. In the (often milder) unoperated children in their series, this was 23 % [13]. The fact that an increased prevalence of these delays is also seen in unoperated children supports the theory that they primarily originate in the brain and might not be a direct result of the craniosynostosis acting as a growth restrictor [3, 4, 52].

## Conclusions

Trigonocephaly is the second most frequent type of craniosynostosis (incidence, 1:5,200) and is associated with a remarkable incidence of intracranial abnormalities and neuropathology. Treatment of the skull malformation consists of a fronto-supraorbital advancement and remodelling, which restores both volume and shape of the skull. The most commonly seen long-term complication after surgery is temporal hollowing.
